# Supplementary material for: The interactome of intact mitochondria by cross-linking mass spectrometry provides evidence for coexisting respiratory supercomplexes
Source: Mol Cell Proteomics. 2017 Dec 8;17(2):216–32. doi: 10.1074/mcp.RA117.000470 (PMC5795388; doi:10.1074/mcp.RA117.000470)
Supplement: Supplemental Data [file supp_RA117.000470_133922_0_supp_23978_fzffwf.zip › spectra_annotation/mito_DR_spectra_annotation/159-1-4-1-10-1.pdf]

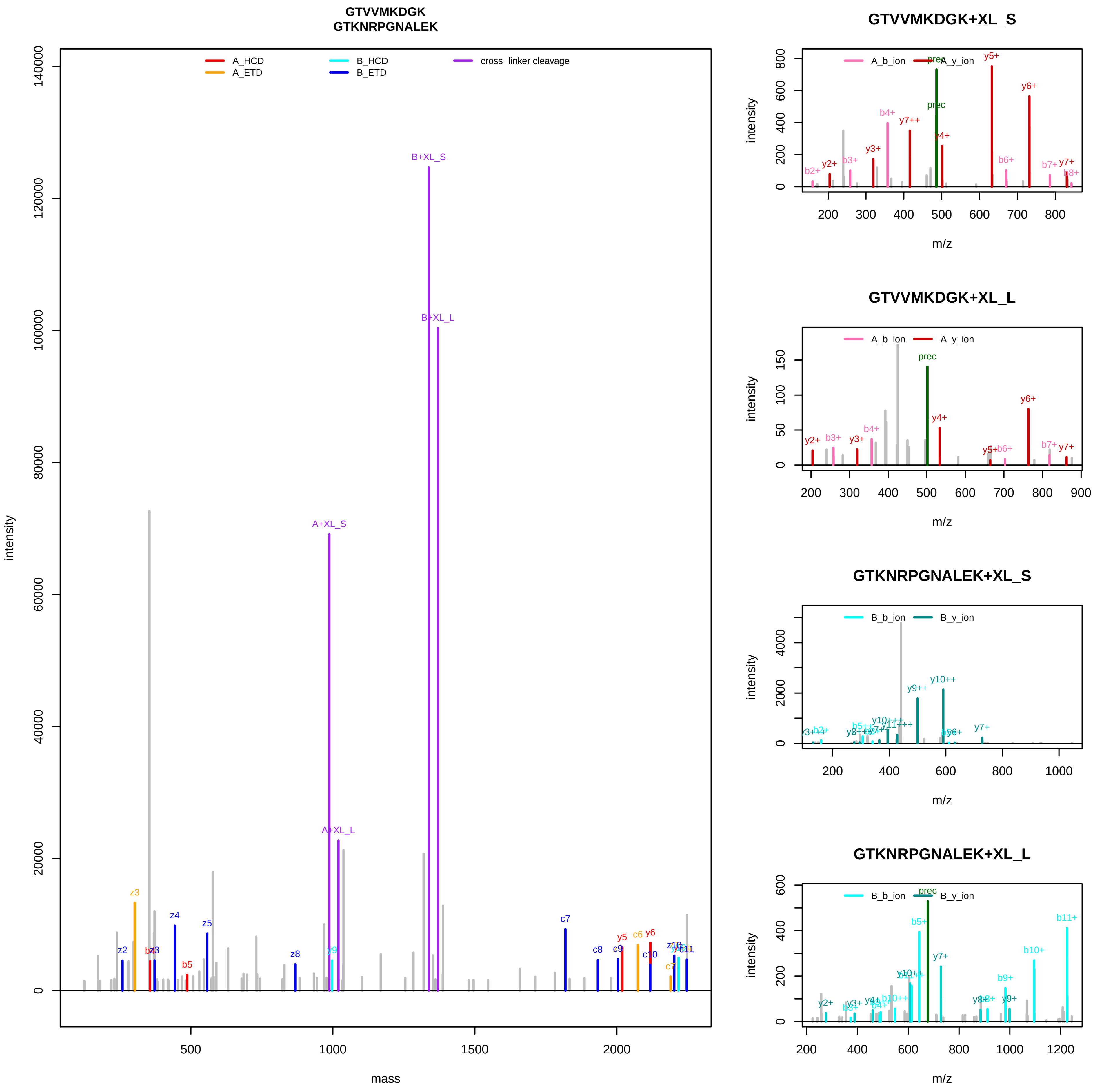
GTVVMKDGK
GTKNRPGNALEK
A_HCD
A_ETD
B_HCD
B_ETD
cross−linker cleavage
B+XL_S
B+XL_L
A+XL_S
A+XL_L
z2
z3
z4
z5
z8
b4
b5
y5
y6
y9
c6
c7
c8
c9
c10
c11
z10
intensity
mass
0
20000
40000
60000
80000
100000
120000
140000
500
1000
1500
2000
GTVVMKDGK+XL_S
A_b_ion
A_y_ion
prec
b2+
b3+
b4+
b6+
b7+
b8+
y2+
y3+
y4+
y5+
y6+
y7+
y7++
m/z
GTVVMKDGK+XL_L
GTKNRPGNALEK+XL_S
B_b_ion
B_y_ion
y9++
y10++
y7+
GTKNRPGNALEK+XL_L
b5+
b10+
b11+
y7+
y8+
y9+
b9+
y2+
